# Amino Alcohols from Eugenol as Potential Semisynthetic Insecticides: Chemical, Biological, and Computational Insights

**DOI:** 10.3390/molecules26216616

**Published:** 2021-10-31

**Authors:** Renato B. Pereira, Nuno F. S. Pinto, Maria José G. Fernandes, Tatiana F. Vieira, Ana Rita O. Rodrigues, David M. Pereira, Sérgio F. Sousa, Elisabete M. S. Castanheira, A. Gil Fortes, M. Sameiro T. Gonçalves

**Affiliations:** 1 REQUIMTE/LAQV, Laboratory of Pharmacognosy, Department of Chemistry, Faculty of Pharmacy, University of Porto, R. Jorge Viterbo Ferreira, 228, 4050-313 Porto, Portugal; rjpereira@ff.up.pt (R.B.P.); dpereira@ff.up.pt (D.M.P.); 2 Centre of Chemistry, Department of Chemistry, University of Minho, Campus of Gualtar, 4710-057 Braga, Portugal; nuno_pinto1993@hotmail.com (N.F.S.P.); mjfernandes@quimica.uminho.pt (M.J.G.F.); gilf@quimica.uminho.pt (A.G.F.); 3 Associate Laboratory i4HB—Institute for Health and Bioeconomy, Faculty of Medicine, University of Porto, 4200-319 Porto, Portugal; tatianafvieira@gmail.com (T.F.V.); segiofsousa@med.up.pt (S.F.S.); 4 UCIBIO—Applied Molecular Biosciences Unit, BioSIM—Department of Biomedicine, Faculty of Medicine, University of Porto, 4200-319 Porto, Portugal; 5 Centre of Physics of Minho and Porto Universities (CF-UM-UP), University of Minho, Campus of Gualtar, 4710-057 Braga, Portugal; ritarodrigues@fisica.uminho.pt (A.R.O.R.); ecoutinho@fisica.uminho.pt (E.M.S.C.)

**Keywords:** eugenol derivatives, amino alcohols, semisynthetic insecticides, biopesticides, bioinsecticides, phenylpropanoids, *Spodoptera frugiperda*

## Abstract

A series of β-amino alcohols were prepared by the reaction of eugenol epoxide with aliphatic and aromatic amine nucleophiles. The synthesized compounds were fully characterized and evaluated as potential insecticides through the assessment of their biological activity against *Sf9* insect cells, compared with a commercial synthetic pesticide (chlorpyrifos, CHPY). Three derivatives bearing a terminal benzene ring, either substituted or unsubstituted, were identified as the most potent molecules, two of them displaying higher toxicity to insect cells than CHPY. In addition, the most promising molecules were able to increase the activity of serine proteases (caspases) pivotal to apoptosis and were more toxic to insect cells than human cells. Structure-based inverted virtual screening and molecular dynamics simulations demonstrate that these molecules likely target acetylcholinesterase and/or the insect odorant-binding proteins and are able to form stable complexes with these proteins. Encapsulation assays in liposomes of DMPG and DPPC/DMPG (1:1) were performed for the most active compound, and high encapsulation efficiencies were obtained. A thermosensitive formulation was achieved with the compound release being more efficient at higher temperatures.

## 1. Introduction

The use of synthetic pesticides for decades to manage pest control in crops has resulted in an accumulation of various residues with adverse effects on many organisms and potential negative impact in human health. At the same time, crop destruction by pests, mainly by insects, is one of the main problems responsible for losses in agricultural production. Pesticides from natural sources are an effective alternative to synthetic pesticides and are becoming more important for pest management in agriculture and also public health. In this respect, plants offer a wide variety of secondary metabolites with efficacy against insects [1,2]. In recent years, essential oils (EOs) became an important natural source of pesticides. Many EOs present insecticidal, repellent, fumigant, and antifeedant activities against a wide variety of insects [3,4]. Essential oil components and their derivatives are considered to be an alternative way of insect control. In particular, phenylpropanoids, one of the main constituents of some EOs, have proved to present efficacy against insects [4]. Eugenol is a phenylpropanoid and a major constituent of clove essential oil with many applications in pharmaceutical, food, agricultural, and cosmetics industries [5], and it has been shown to be biologically active as antioxidant [5,6], antiviral [7] anti-inflammatory [8] and antimicrobial [9]. Enan [10] showed that eugenol mimicked octopamine in increasing intracellular calcium levels in cloned cells from the brain of *Periplaneta americana* and *Drosophila melanogaster*, and this was also found to be mediated via octopamine receptors. Structural changes of eugenol are known to be a useful strategy in order to improve biological activity and to obtain new analogues with reduced side effects [11].

Further, epoxides are important intermediates in pharmaceutical and agrochemical industries. The three-membered heterocyclic ring is strained and susceptible to attack by a range of nucleophiles, including nitrogen (e.g., ammonia, amines, azides), oxygen (e.g., water, alcohols, phenols, acids), and sulfur (thiol)-containing compounds, leading to bifunctional molecules of great industrial value. The β-amino alcohols are used in the synthesis of β-blockers, insecticidal agents, and oxazolines, as well as chiral ligands in asymmetric synthesis [12,13,14,15,16,17]. β-Amino alcohol functionality is found in many biologically active compounds, being an important pharmacophore [14,18], and *N*-substituted β-amino alcohols are important building blocks in the preparation of added-value chemicals [16,19]. Salbutamol and propranolol are on the World Health Organization List of Essential Medicines and represent the most important examples of therapeutic agents having this structural feature [20].

Following our research interests in plant-inspired alternatives to synthetic pesticides [21,22], and specifically the study in which some eugenol derivatives have shown potential as biopesticides [22], in the present work, eugenol was converted to the corresponding epoxide with *m*-chloroperoxybenzoic acid (*m*-CPBA) in dichloromethane (DCM) and further reacted with a series of amine nucleophiles to afford the corresponding β-amino alcohols. The β-amino alcohols were purified by column chromatography and fully characterized by ^1^H and ^13^C NMR spectroscopy and HRMS (high-resolution mass spectrometry). The obtained compounds were evaluated as potential insecticides through the assessment of their biological activity against the *Sf9* insect cell lines compared with chlorpyrifos, which is a commercial synthetic pesticide. In addition, computational studies were performed to identify the most likely protein targets responsible for the observed insecticide activity of these molecules through the application of structure-based inverted virtual screening protocol combined with molecular dynamics simulations and free energy calculations. Nanoencapsulation of the most promising compound considering its insecticidal activity was performed in liposomes of DMPG (dimyristoylphosphatidylglycerol) and DPPC/DMPG (dipalmitoylphosphatidylcholine/dimyristoylphosphatidylglycerol) (1:1), aiming at obtaining thermosensitive formulations. DMPG has a gel to liquid–crystalline phase transition temperature (T_m_) at 23 °C, while for DPPC, it is 41 °C [23]. The increase in membrane fluidity upon phase transition is expected to promote an enhanced release of the encapsulated compounds, providing a triggered release by temperature above T_m_ of the formulation.

## 2. Results and Discussion

### 2.1. Synthesis

**Scheme 1:**
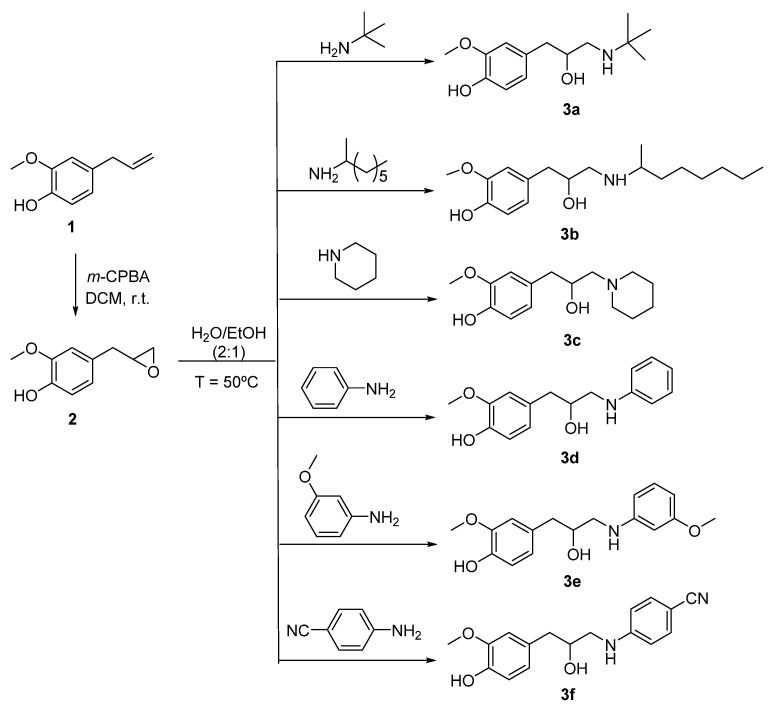
Synthesis of eugenol amino alcohols **3a**–**f**.

Eugenol **1** is easily obtained by hydrodistillation from clove, and is known for its various biological activities, as mentioned above, namely insecticidal. Following our recent interests in finding new biopesticides [22], the present work describes a strategy consisting of structural changes of eugenol in an attempt to obtain semisynthetic alternatives with improved insecticidal activity. Eugenol epoxide **2** was prepared from eugenol through reaction with *m*-CPBA in DCM using a known procedure [11,24] in 48% yield. Then, the epoxide was further reacted at 50 °C with a series of aliphatic and aromatic amine nucleophiles in ethanol/water as solvent [25], which is followed by column chromatography purification on silica gel using dichloromethane/methanol, mixtures of increasing polarity (**3b**, **3d–f**) as the eluent, or by evaporation of solvents under reduced pressure (**3a** and **3c**) to afford the corresponding β-amino alcohol derivatives **3a**–**f** as oil materials. Thus, the reaction of 2-methoxy-4-(oxiran-2-ylmethyl)phenol **2** with 2-methylpropan-2-amine, octan-2-amine, piperidine, aniline, 3-methoxyaniline, and 4-cyanoaniline gave 4-(3-(*tert*-butylamino)-2-hydroxypropyl)-2-methoxyphenol **3a**, 4-(2-hydroxy-3-(octan-2-ylamino)propyl)-2-methoxyphenol **3b**, (4-(2-hydroxy-3-(piperi- din-1-yl)-propyl)-2-methoxyphenol **3c**, 4-(2-hydroxy-3-(methyl(phenyl)amino)propyl)- 2-methoxyphenol **3d**, 4-(2-hydroxy-3-((3-methoxyphenyl)amino)propyl)-2-methoxy- phenol **3e**, and 4-(2-hydroxy-3-(4-hydroxy-3-methoxyphenyl)propyl)amino)benzonitrile **3f**, respectively, in 9–97% yield, and they were fully characterized by ^1^H and ^13^C NMR spectroscopy and HRMS (Scheme 1).

The main ^1^H-NMR features of compounds **3a**–**f** are the signals for protons of the CHOH, CH_2_N, and OCH_3_ groups. The CH_2_N protons show up as two distinct signals as doublets of doublets or multiplets (δ 3.45–2.53 ppm); the CHOH proton displays as a multiplet in all compounds (δ 4.91–3.89 ppm); and the OCH_3_ group shows up as a singlet (δ 3.89–3.81 ppm). The *tert*-butyl group in **3a** shows up as a singlet (δ 1.16 ppm), while in **3b** and **3c**, the *N*-alkyl chain corresponds to a series of multiplets (δ 2.88–0.88 ppm). The ^13^C main features are the CH_2_N (δ 41.24–41.10 ppm), CHOH carbon (δ 71.1–67.01 ppm), OCH_3_ carbon (δ 55.93–55.72 ppm), and the additional OCH_3_ in **3e** (δ 54.96 ppm). In addition, the *tert*-butyl carbons in **3a** shows up (δ 26.79 and 24.58 ppm), while in **3b**, the methyl terminal is highlighted (δ 13.98 ppm).

### 2.2. Toxicity Assessment in Insect Cells

**Figure 1:**
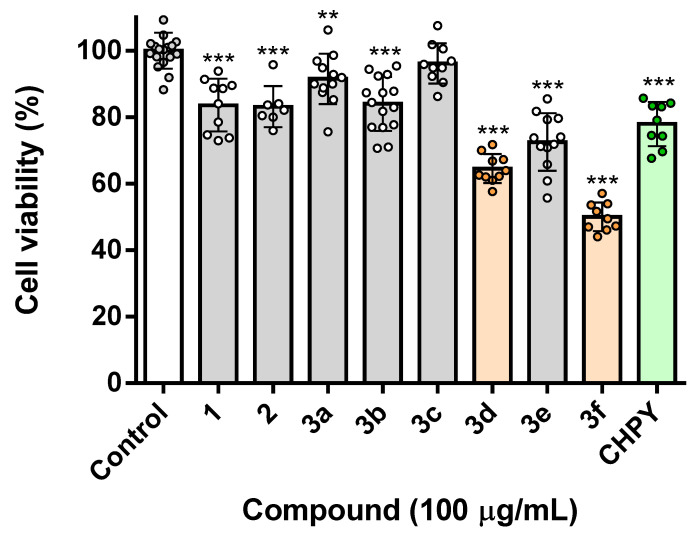
Viability of the *Sf9* cells after incubation with the presented molecules (100 µg/mL), medium (control), or the reference insecticide chlorpyrifos (CHPY, 100 µg/mL). Cells were incubated for 24 h, after which viability was evaluated. ** *p* < 0.01, *** *p* < 0.001.

All molecules obtained were evaluated for their impact in the viability of the *Sf9* cells at 100 µg/mL (i.e., **1**—6.09 × 10^−4^ M; **2**—5.55 × 10^−4^ M; **3a**—3.95 × 10^−4^ M; **3b**—3.23 × 10^−4^ M; **3c**—3.77 × 10^−4^ M; **3d**—3.66 × 10^−4^ M; **3e**—3.30 × 10^−4^ M; **3f**—3.35 × 10^−4^ M; **CHPY**–2.85 × 10^−4^ M) by the means of a resazurin-based method. For benchmarking purposes, the insecticide chlorpyrifos was used at the same concentration. As shown in Figure 1, the only molecule devoid of toxicity was **3c**, which incidentally was also the only one bearing a piperidine ring. A second group of molecules, which elicited residual toxicity (under 25% of viability loss), was **1**, **2**, **3a**, and **3b**. Eugenol **1** was the starting material, and the results show that the replacement of the terminal methylene group by the epoxide had no effect upon the biological activity of the molecule. Finally, the most potent molecules were **3d**, **3e**, and **3f**, which caused losses of 40%, 30%, and 50% viability in insect cells, respectively. These three molecules were also the only ones bearing a benzene ring next to the nitrogen atom. Considering the unsubstituted ring, **3d**, its methoxylation resulted in decreased potency, while the presence of the cyanide group increased it. In light of these results, we decided to advance our studies solely with the two most potent molecules, **3d** and **3f**, as they were more potent than the benchmark used, chlorpyrifos.

### 2.3. Amino Alcohols ***3d*** and ***3f*** Activate Caspase-like Proteases in the Sf9 Cells

**Figure 2:**
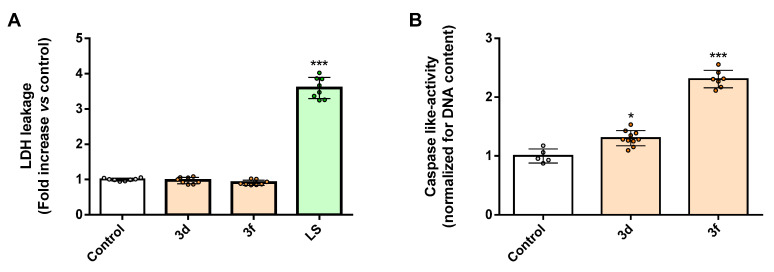
(**A**) LDH activity found in the culture media of the *Sf9* cells treated with compounds **3d** and **3f** (100 µg/mL) for 24 h. Lysis solution (LS) was used as positive control to generate a maximum LDH release. (**B**) Caspase-like activity of the *Sf9* cells after incubation with compounds **3d** and **3f** (100 µg/mL) for 24 h. Results are normalized for DNA content. * *p* < 0.05, *** *p* < 0.001.

After establishing the toxicity of the selected molecules toward insect cells, we investigated the mechanism of action behind this effect. In fact, the loss of viability could be a consequence of an array of different biological processes, from necrosis to cell cycle arrest and apoptosis, among others. Necrosis is a process of uncontrolled cell death that encompasses the destruction of cell membranes, with consequent leakage of cytoplasmic content to the surrounding tissues; for this reason, it is usually avoided in biological contexts [26]. To assess the potential unfolding of this event, we assessed the levels of leaked lactate dehydrogenase (LDH) in cells incubated with the selected molecules. Being a cytoplasmic enzyme, the finding of extracellular LDH is widely used as a marker of necrosis. As shown in Figure 2A, the incubation of cells with a lysis solution (LS) resulted in a three to four-fold increase in extracellular LDH. Conversely, the incubation of cells with **3d** and **3f** had no detectable impact in LDH levels in culture media. In light of this, we concluded that the impact of these molecules in the viability of the *Sf9* cells was not a consequence of an ongoing necrotic process. Next, we assessed if a process of organized cell death, such as apoptosis, could be taking place. Given the pivotal role of cysteine-aspartic proteases in most forms of apoptosis, we investigated the impact of **3d** and **3f** in the insect equivalent of mammal caspases, in this case DRACE, using a proluminescent substrate of this target. As shown in Figure 2B, both **3d** and **3f** significantly increased the caspase-like activity in treated cells, the latter having a more pronounced effect. This result suggests that both **3d** and **3f** elicit their cytotoxic effect toward the *Sf9* cells by triggering an organized process of cell death with the involvement of cysteine-aspartic proteases.

### 2.4. Amino Alcohols ***3d*** and ***3f*** Are More Toxic to Insect Cells Than Human Cells

**Figure 3:**
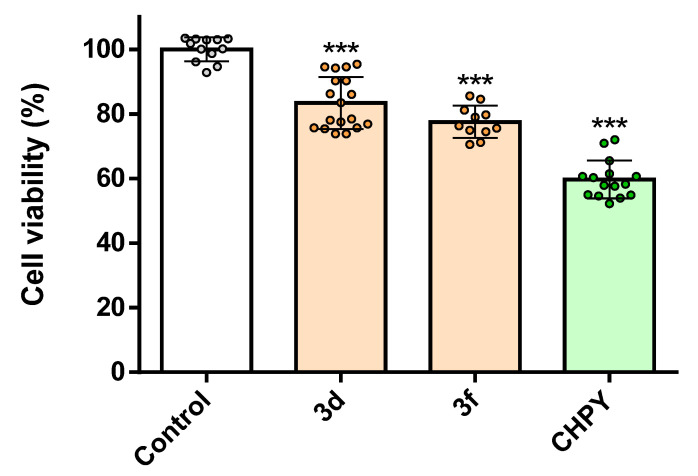
Viability of HaCaT cells exposed to compounds **3d** and **3f** (100 µg/mL), medium (control), or the reference insecticide chlorpyrifos (CHPY, 100 µg/mL). Cells were incubated for 24 h, after which viability was evaluated. *** *p* < 0.001.

Up to this point, we had already identified two molecules that presented higher potency than the commercial insecticide chlorpyrifos and that were shown the be non-necrotic and pro-apoptotic. In addition to these traits reported herein, it is also important that prospective new insecticides present some degree of selectivity, specifically low toxicity to human cells. To this end, we assessed the impact of the **3d** and **3f** in 2D models of human cells. We chose human keratinocytes (HaCaT cell line), as one of the most relevant routes of human contact with pesticides is usually via the skin, where keratinocytes are the first population of living cells in the skin. As shown in Figure 3, both molecules elicited a weak loss of viability, around 20%. Importantly, both molecules were less toxic than the benchmark chlorpyrifos and, relevantly, they were less toxic to human cells than insect cells.

These results are promising and pave the way for further developments in the field, as the chemical diversity obtained allows drawing some structure–activity relationships, as addressed above.

### 2.5. Inverted Virtual Screening Results

After identifying the most promising molecules and the biological processes involved in their cytotoxic effect, we were interested in shedding light on the possible molecular targets. To this end, an array of computational methods was used.

Table 1 presents the average scores obtained for compounds **3d** and **3f** for each potential target with each scoring function. Regarding the different scoring functions, it is important to mention that they are based on different metrics and scales. The score for all the GOLD scoring functions is dimensionless, and the higher the score, the better the binding affinity. The Vina scoring function, on the other hand, uses a metric that approximates that of binding free energies, so a more negative value means better affinity. The PDB structure with the best score was selected for each potential target, and they were ranked from the best target to worst, according to the predictions of the different docking programs/scoring functions.

Globally, considering the results obtained with the different scoring functions, the odorant binding proteins class (OBP) and acetylcholinesterase (AChE) are the protein targets with the highest affinity toward compounds **3d** and **3f**. This tendency was quite clear with all the different scoring functions evaluated, which further strengthens our results.

### 2.6. Molecular Dynamics Simulations and Free Energy Calculations Results

To validate the inverted screening results, we evaluate the protein flexibility and characterize the molecular interactions formed, and molecular dynamics simulations were performed for the complexes formed with compound **3d** and compound **3f** and the two groups of targets predicted at the inverted VS stage: OBP and AChE. Structures with the best score from each group were selected (3K1E for OBP and 1QON for AChE). The stability of AChE: compound **3d**, AChE: compound **3f**, OBP: compound **3d**, and OBP: compound **3f** complexes was evaluated using RMSD calculations for the Cα atoms of each complex and for the ligands, Solvent-Accessible Surface Area (SASA) analysis, and hydrogen bonding analysis.

All systems and ligands presented relatively low RMSD values, as seen in Table 2 (and Figure S1), showing that the target–ligand complexes are well equilibrated and that the eugenol derivatives evaluated maintain their binding conformation predicted from the docking.

When analyzing the percentage of potential SASA area buried for compound **3d** and compound **3f** when complexed with AChE and OBP, it can be seen that the two molecules remain tightly bound to the two targets evaluated and well protected from the solvent with average buried areas over 90% (Table 2). A small decrease was noticed for compound **3d** bound to AChE, in relation to the initial configuration predicted from docking, with an average buried area oscillating between 80 and 90%. These results demonstrate that compounds **3d** and **3f** remain well bound to the two targets evaluated, even after 100 ns. In particular, the eugenol derivatives evaluated in complex with OBP remain very well protected from the solvent throughout time.

Hydrogen bonding analysis is important to understand the stability of the interactions between the targets and ligands throughout time. The results presented in Table 2 show that both ligands maintain a stable hydrogen bonding profile with the targets evaluated, maintaining between one and three hydrogen bonds with AChE and one and four hydrogen bonds with OBP. Globally, the profile observed shows that compounds **3d** and **3f** establish more hydrogen bonds with OBP and with AChE.

Table 2 summarizes the results discussed so far and presents the values for the Gibbs binding free energy calculated using MM/GBSA. The analysis of the residue contribution to the eugenol derivatives’ binding free energy to the two protein targets evaluated highlights the interaction profile of compounds **3d** and **3f** against AChE and OBP, showing the most important amino acid residues involved in ligand stabilization.

AChE is a serine hydrolase, and it is a very common target for pesticides as it is an enzyme vital for the regulation of acetylcholine in several organisms, from insects to mammals. Since this is an enzyme transversal to many species, the use of anticholinesterase insecticides can cause serious health and environmental problems. In addition, there are reports of insect resistance due to mutation of the AChE gene [27].

**Figure 4:**
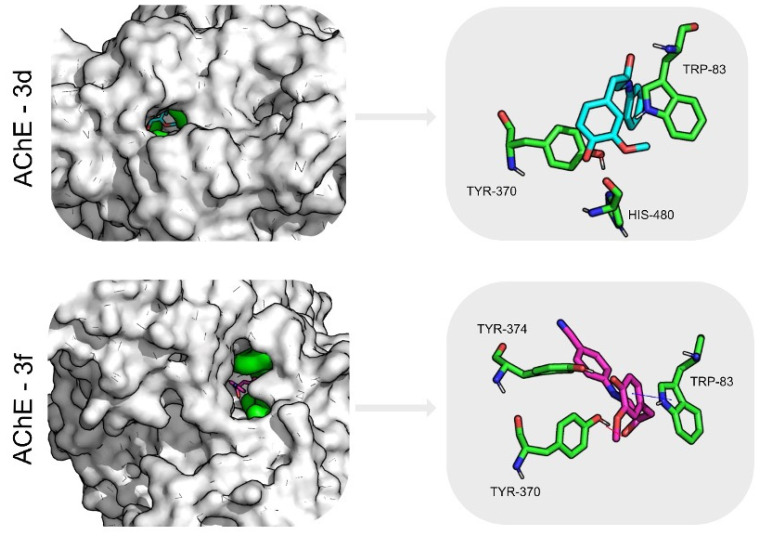
Compound **3d** (cyan licorice) and compound **3f** (pink licorice) interaction map with AChE. The most important residues for the interaction are highlighted in green. Blue arrow indicates π–π stacking with the ring of Trp-83. Red lines represent hydrogen bonding.

For AChE, compound **3d** binding is stabilized mostly by residues Trp83 (−2.4 ± 0.8), Tyr370 (−1.3 ± 0.4), and His480 (−1.3 ± 0.6) through non-polar interactions. For compound **3f**, the residues contributing more toward AChE binding are Tyr370 (−2.4 ± 0.1), Tyr372 (−2.5 ± 0.8), and Trp83 (−1.9 ± 0.4), with non-polar interactions playing an important role and π–π stacking with Trp83. Figure 4 represents the average structure of the dominant cluster of the AChE-eugenol derivatives complexes obtained from the analysis of the MD trajectory, illustrating the binding pocket and main interactions formed.

For the sake of warranting the potential off-target effect that could pose a toxicity risk, human AChE was also analyzed, the docking scores (Table S1) being inferior to the ones obtained for insect AChE, hence suggesting that the eugenol derivatives evaluated favor binding to the insect AChE considered over that of human AChE. When comparing the sequence of amino acids between insect and human AChE, there is only 53–54% sequence identity, even though their 3D structures are very similar. The active-site gorge in the insect enzyme is narrower, and the amino acid residues are different. Moreover, the residues in the opening of the gorge are also different [27], which might explain the difference in affinity of the eugenol derivatives.

The results show that the most stable complexes are OBP–compound **3d** and OBP–compound **3f**, with binding free energy values of −31.7 ± 0.2 and −41.6 ± 0.2 kcal/mol, respectively. This is consistent with the results presented so far and indicates that eugenol derivatives have indeed a high affinity toward OBP. In fact, there is a structure deposited in the PDB of a bee OBP14 from bound to eugenol [28].

OBP are a large and diverse family of insect proteins. They are involved in the transport of hydrophobic odorant and pheromone molecules toward the olfactory receptors. They are abundant in the insect family and different in structure but carry out similar roles. In the *Drosophila melanogaster*, there are 52 different types of OBPs alone. Even though diverse in number and sequence, they present some common features. They are small, have six conserved cysteine residues joined by three disulfide bridges, and have six alpha-helical domains [29,30,31,32].

Compounds **3d** and **3f** have a higher molecular weight than eugenol (273.33 g/mol, 298.34 g/mol, respectively, versus 164.20 g/mol), but they are also lipophilic and if volatile, they can in fact be capable of binding OBP. The precise mechanism of action still needs to be further validated.

**Figure 5:**
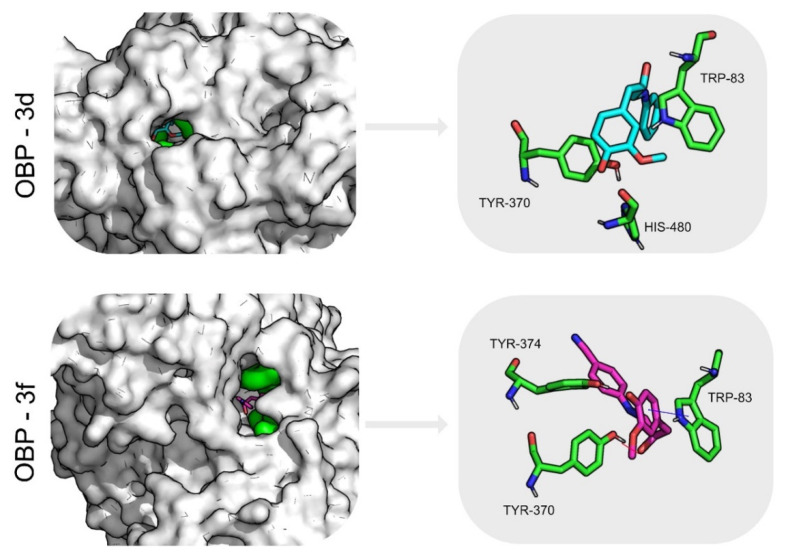
Compound **3d** (cyan licorice) and compound **3f** (pink licorice) interaction map with OBP1. The most important residues are highlighted in green.

For OBP1, compound **3d** binding is stabilized mostly by residues Leu67 (−2.5 ± 0.5), Trp105 (−2.1 ± 0.4), and Ala79 (−1.7 ± 0.5), through van der Waals interactions. For compound **3f**, the residues contributing more toward OBP binding are Met75 (−2.9 ± 0.4) and Phe114 (−1.8 ± 0.8) through van der Waals interactions and Trp105 (−2.5 ± 0.4) through a hydrogen bond with the backbone. Figure 5 illustrates the average structure of the dominant cluster of the OBP1 binding pocket and main interactions formed between compound **3d**-OBP1 and compound **3f**-OBP1, respectively.

### 2.7. Nanoencapsulation and Release Assays

The most active compound against the *Sf9* cells, compound **3f**, was encapsulated in liposomal systems of the phospholipids DMPG (100%) and DMPG/DPPC (1:1). The size (hydrodynamic diameter), polydispersity index, and zeta potential of the compound-loaded liposomes were determined by Dynamic and Electrophoretic Light Scattering (Table 3). These properties can affect the bulk properties, performance, processability, and stability of a nanoformulation. Particularly, the surface charge highly influences the stability of the liposomes, and zeta potential values more negative than −30 mV or more positive than +30 mV are considered optimal values for good stabilization of a nanodispersion [33]. In view of this fact, a negatively charged lipid, phosphatidylglycerol, was chosen for the liposomal formulation. Specifically, the phospholipid DMPG has a phase transition temperature (23 °C) near room temperature [23], allowing obtaining an enhanced release at summer temperatures (around or above 30 °C), where the lipid is in the fluid (liquid-crystalline) phase. However, the relatively short hydrocarbon chain of DMPG and the tendency to form leaky vesicles [34] can hamper a high encapsulation efficiency of compound **3f**. Therefore, the DPPC/DPPG (1:1) formulation was also tested.

The values in Table 3 show that both liposome formulations are small in size, with hydrodynamic diameters around 200 nm, presenting also a low polydispersity. A PDI value below 0.3 is considered to be acceptable, indicating a homogenous population of phospholipid vesicles [35]. The zeta potential values indicate a highly negative surface charge, anticipating a low aggregation (due to the electrostatic repulsion) and high colloidal stability.

**Figure 6:**
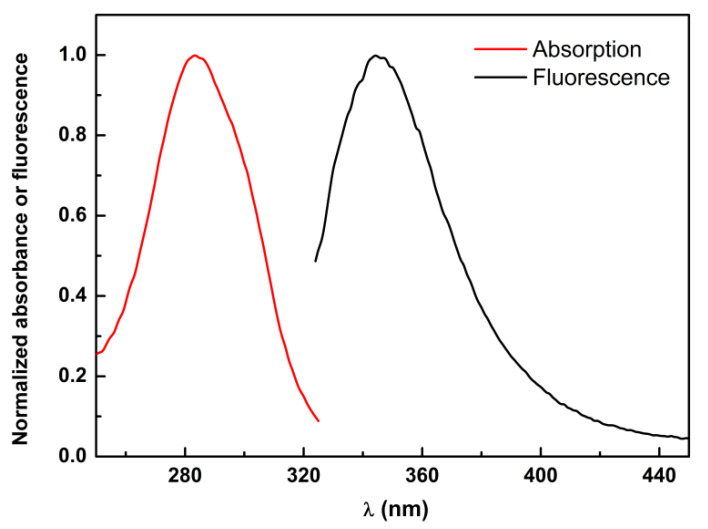
Normalized absorption and fluorescence emission (excitation at 290 nm) spectra of **3f** solution (2 × 10^−5^ M for absorption and 5 × 10^−6^ M for emission).

The compound **3f** is a fluorescent molecule (Figure 6) in several solvents and in liposomes. This is a great advantage for the determination of the encapsulation efficiency and drug release, due to the high sensitivity (and selectivity) of fluorescence spectroscopy.

The encapsulation efficiency (EE%) of compound **3f** in liposomes was determined by fluorescence measurements (Table 4). In these assays, it was kept in mind that the compound is active against the *Sf9* cells at a concentration of 100 µg/mL (3.35 × 10^−4^ M) and, therefore, at least this concentration must be encapsulated. The encapsulation efficiencies are high, the system DPPC/DMPG being the most advantageous for **3f** encapsulation. Nevertheless, the EE% values show that both formulations are able to encapsulate **3f** at concentrations that may guarantee an insecticidal activity (if compound release is effective).

**Figure 7:**
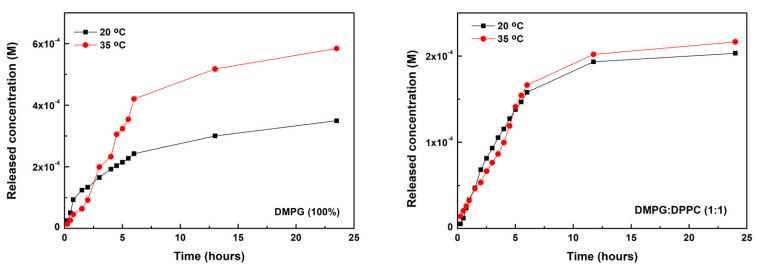
Cumulative release of compound **3f** at 20 °C (black squares) and 35 °C (red dots) from liposomes of DMPG (100%) (**left**) and DMPG:DPPC (**right**).

Compound release from both liposomal formulations was studied at 20 °C and 35 °C, to investigate the temperature dependence of the release profile (Figure 7). The experimental data were analyzed with the Weibull model (Table S2 and Figure S4 in Supporting Information) and the cumulative concentration released was compared in terms of liposome formulation and temperature.

An enhanced release of **3f** was observed from liposomes of DMPG, reaching a cumulative release of 62% in 24 h at 35 °C, while, at 20 °C, a 36% release was attained. This is a result of the higher membrane fluidity at 35 °C (above transition temperature) for DMPG liposomes, which provide a thermosensitive formulation. Moreover, at both temperatures, the compound released is higher than 100 µg/mL in 24 h. The rigidity of DPPC at temperatures below its gel to liquid-crystalline phase transition (41 °C) justifies the much lower compound release from DPPC/DMPG liposomes, with 14% and 16% of released compound at 20 °C and 35 °C, respectively, not displaying a significant sensitivity to temperature.

The parameter *b* of the Weibull model can be related to the release mechanism [36]. If *b* ≤ 0.75, the release is due to a Fickian diffusion, which is the case for DMPG liposomes at 20 °C (Table S2). If *b* > 1, a complex release mechanism takes place, with multiple mechanisms contributions, which is verified in the other cases.

## 3. Materials and Methods

### 3.1. Chemicals and Reagents

Dichloromethane, ethanol, methanol, ethyl acetate, light petroleum, and *m*-chloroperbenzoic acid were purchased from Fisher Scientific (Geel, Belgium). The anhydrous magnesium sulfate was PanReac Applichem (Barcelona, Spain) products. Chloroform-d was produced by Eurisotop (Cambridge, England). Thin-layer chromatography (TLC) analyses were carried out on 0.25 mm thick, precoated silica plates (Merck Fertigplatten Kieselgel 60F254, Germany), and spots were visualized under UVlight. Chromatography on silica gel was carried out on Merck Kieselgel (230–240 mesh).

### 3.2. Analytical Instruments

NMR spectra were obtained on a Bruker Avance III (Bruker Corporation, Billerica, MA, USA) at an operating frequency of 400 MHz for ^1^H NMR and 100.6 MHz for ^13^C NMR using the solvent peak as internal reference at 25 °C. All chemical shifts are given in ppm using δ Me_4_Si = 0 ppm as reference, and J values are given in hertz. Assignments were made by comparison of chemical shifts, peak multiplicities, and J values and were supported by spin decoupling-double resonance and bidimensional heteronuclear correlation techniques. High-resolution mass spectrometry analyses were performed at the “CACTI—Unidade de Masas e Proteómica, at University of Santiago de Compostela”, Spain.

### 3.3. Synthesis of 2-Methoxy-4-(oxiran-2-ylmethyl)phenol ***2***

A solution of eugenol **1** (0.500 g 3.0 mmol; 1 equiv) dissolved in dichloromethane (18 mL) was added dropwise to a suspension of 70% *m*-chloroperbenzoic acid (0.750 g; 4.3 mmol; 1 equiv) in dichloromethane (10 mL) at 0 °C. After stirring for 1 h, *m*-chloroperbenzoic acid was again added (1 equiv), and the reaction mixture was stirred for another 24 h at room temperature. A 10% aqueous solution of sodium sulfate (2 × 20 mL) was added, and the resulting solution was washed with 5% aqueous solution of sodium hydrogen carbonate (2 × 20 mL). The organic phase was dried with anhydrous magnesium sulfate, and the solvent was evaporated to afford the compound **2** as a dark yellow oil (0.239 g; 48%). Rf = 0.27 (DCM). ^1^H-NMR δ_H_ (CDCl_3_, 400 MHz): 6.87 (d, 1H, J = 8 Hz, H-6), 6.73–6.78 (m, 2H, H-3 and H-5), 5.54 (s, 1H, OH), 3.90 (s, 3H, OCH_3_), 3.12–3.16 (m, 1H, CH oxirane), 2.79–2.82 (m, 3H, CH_2_Ph and CH_2_ oxirane), 2.55 (q, J = 2.8 Hz, 1H, CH_2_ oxirane) ppm. ^13^C-NMR δ_C_ (CDCl_3_, 100.6 MHz): 146.46 (C-2), 144.39 (C-1), 129.03 (C-4), 121.64 (C-5), 114.32 (C-6), 111.54 (C-3), 55.90 (OCH_3_), 52.67 (CH oxirane), 46.79 (CH_2_ oxirane), 38.37 (CH_2_Ph) ppm.

### 3.4. Synthesis of Amino Alcohols ***3a–f***

#### 3.4.1. Synthesis of 4-(3-(Tert-butylamino)-2-hydroxypropyl)-2-methoxyphenol **3a**

To a suspension of 2-methoxy-4-(oxiran-2-ilmethyl)phenol **2** (0.163 g; 0.90 mmol; 1 equiv) in H_2_O/EtOH 2:1 (2 mL) was added 2-methylpropan-2-amine (0.325 g; 4.44 mmol), and the resulting mixture was heated at 50 C for 5 h. The solvents and the amine were evaporated under reduced pressure to afford a compound **3a** as an orange oil (0.097 g; 0.38 mmol; 42%), Rf = 0.30 (MeOH/DCM 1:9). ^1^H-NMR δ_H_ (CDCl_3_, 400 MHz): 6.78 (d, J = 8.0 Hz, 1H, Ar-H), 6.74 (d, J = 1.6 Hz, 1H, Ar-H), 6.45 (dd, J = 8.4 Hz, 2.0 Hz 1H, Ar-H), 4.04–3.98 (m, 1H, CH_2_CH(OH)), 3.81 (s, 3H, OCH_3_), 3.45 (s, 1H, CH_2_NH), 2.81 (dd, J = 12.0 Hz, 2.4 Hz, 1H, CH_2_NH), 2.77–2.62 (m, 2H, CH_2_CH(OH)), 1.16 (s, 9H, *t*-Bu) ppm. ^13^C-NMR δ_C_ (CDCl_3_, 100.6 MHz): 146.63 (Ar-C),144.34 (Ar-C), 121.83 (Ar-C), 114.4 (Ar-C), 112.13 (Ar-C), 69.37 (CH_2_CH(OH)), 55.8 (OCH_3_), 47.06 (CH_2_), 41.25 (CH_2_), 26.79 (3×CH_3_), 24.58 (C(CH_3_)) ppm. HRMS (ESI-TOF): calcd for C_14_H_24_NO_3_ [M ^+^ + H]: 254.1751; found 254.1753.

#### 3.4.2. Synthesis of 4-(2-Hydroxy-3-(octan-2-ylamino)propyl)-2-methoxyphenol **3b**

To a suspension of 2-methoxy-4-(oxiran-2-ilmethyl)phenol **2** (0.163 g; 0.90 mmol; 1 equiv) in H_2_O/EtOH 2:1 (2 mL) was added octan-2-amine (0.502 g; 3.89 mmol) and the resulting mixture was heated at 50 °C for 4 h. Then, water (2 mL) was added, and the resulting mixture extracted with EtOAc (2 mL), The organic phase was collected, dried with anhydrous MgSO_4_, and the solvent evaporated to afford an oil (0.202 g), which was subjected to column chromatography using DCM/MeOH as eluent of increasing polarity to give the compound **3b** as a brown oil (0.165 g; 0.53 mmol; 59%). Rf = 0.45 (MeOH/DCM 10:90). 1H-NMR δ_H_ (CDCl_3_, 400 MHz): 6.78 (d, J = 8.0 Hz, 1H, Ar-H), 6.72 (ls, 1H, Ar-H), 6.48 (d, J = 8.0 Hz, 1H, Ar-H), 3.92–3.89 (m, 1H, CH_2_CH(OH)), 3.81 (s, 3H, OCH_3_), 2.84–2.53 (m, 6H, 2×CH_2_ and CH_2_NH), 1.49 (m, 1H, NHCHCH_3_), 1.24 (m, 10H, 5×CH_2_), 1.07 (m, 3H, NHCHCH_3_), 0.88 (m, 3H, CH_2_CH_2_CH_3_) ppm. ^13^C-NMR δ_C_ (CDCl_3_, 100.6 MHz): 146.69 (Ar-Cq),144.36 (Ar-Cq), 129.59 (Ar-Cq), 121.76 (Ar-C), 114.55 (Ar-C), 111.99 (Ar-C), 70.25 (CH), 69.93 (CH), 55.72 (OCH3), 51.48 (CH_2_), 41.32 (CH_2_), 35.61 (CH_2_), 31.68 (CH_2_), 29.21 (CH_2_), 25.77 (CH_2_), 22.51 (CH_2_), 19.17 (CH_3_), 13.98 (CH_3_) ppm.

#### 3.4.3. Synthesis of 4-(2-Hydroxy-3-(piperidin-1-yl)propyl)-2-methoxyphenol **3c**

To a suspension of 2-methoxy-4-(oxiran-2-ilmethyl)phenol **2** (0.1 g; 0.56 mmol; 1 equiv) in H_2_O/EtOH 2:1 (2 mL) was added piperidine (0.047 g; 0.56 mmol), and the resulting mixture was heated at 50 °C for 5 h. The solvent was evaporated under reduced pressure to afford compound **3c** as a brown oil (0.142 g; 0.54 mmol; 97%). Rf = 0.35 (MeOH/DCM 10:90). ^1^H-NMR δ_H_ (CDCl_3_, 400 MHz): 6.83 (d, J = 8.4 Hz, 1 H, Ar-H), 6.77 (d, J = 2.0 Hz, 1H, Ar-H), 6.67 (dd, J = 8.0 Hz, 2.0 Hz, 1H, Ar-H), 4.18–4.12 (m, 1H, CH_2_CH(OH)), 3.89 (s, 3H, OCH_3_), 2.88–2.81 (m, 4H, CH_2_ and CH_2_NH), 2.65–2.52 (m, 4H, 2×CH_2_), 1.82–1.70 (m, 4H, 2×CH_2_) 1.58–1.47 (m, 4H, 2×CH_2_) ppm. ^13^C-NMR δ_C_ (CDCl_3_, 100.6 MHz): 146.52 (Ar-Cq), 144.31 (Ar-Cq), 129.52 (Ar-Cq), 121.80 (Ar-C), 114.27 (Ar-C), 111.80 (Ar-C), 67.01 (CH), 63.93 (CH_2_), 55.93 (OCH_3_), 54.76 (CH_2_), 41.30 (CH_2_), 24.26 (CH_2_), 23.05 (CH_2_) ppm. HRMS (ESI-TOF): calcd for C_15_H_24_NO_3_ [M ^+^ +H]: 266.1751, found 266.1752.

#### 3.4.4. Synthesis of 4-(2-Hydroxy-3-(phenylamino)propyl)-2-methoxyphenol **3d**

To a suspension of 2-methoxy-4-(oxiran-2-ilmethyl)phenol **2** (1 equiv) in H_2_O/EtOH 2:1 (2 mL) was added aniline (0.4 mL; 3.9 equiv), and the resulting mixture was heated at 50 °C for 5.5 h. Then, water (2 mL) was added, and the resulting mixture was extracted with EtOAc (2 mL). The organic phase was collected, dried with anhydrous MgSO_4_, and the solvent was evaporated to afford an oil (0.284 g), which was subjected to column chromatography using DCM/MeOH as eluent of increasing polarity to give compound **3d** as a yellow oil (0.095 g; 0.35 mmol; 36%). Rf = 0.7 (MeOH/DCM 5:95). ^1^H-NMR δ_H_ (CDCl_3_, 400 MHz): 7.19 (t, J = 7.2 Hz, 2 H, Ar-H), 6.88 (d, J = 8 Hz, 1H, Ar-H), 6.75 (m, 3H, Ar-H), 6.65 (d, J = 8 Hz, 2H, Ar-H), 4.09–4.02 (m, 1H, CH_2_CH(OH)), 3.87 (s, 3H, OCH_3_), 3.31 (dd, J = 12.4 Hz, 7.2 Hz, 1H, CH_2_NH), 3.10 (dd, J = 12.4 and 8 Hz, 1H, CH_2_NH), 2.83 (dd, J = 14 Hz and 5.2 Hz, 1H, CH_2_CH(OH)), 2.75 (dd, J = 14 and 8 Hz, 1H, CH_2_CH(OH)) ppm. ^13^C-NMR δ_C_ (CDCl_3_, 100.6 MHz): 147.9 (Ar-C), 146.6 (Ar-C), 144.4 (Ar-C), 129.4 (Ar-C), 129.3 (Ar-C), 122.0 (Ar-C), 118.1 (Ar-C), 114.5 (Ar-C), 113.5 (Ar-C), 111.8 (Ar-C), 71.1 (CH), 55.9 (OCH_3_), 49.5 (CH_2_), 41.2 (CH_2_) ppm HRMS (ESI-TOF): calcd for C_16_H_20_NO_3_ [M^+^ +H]: 274.1438; found 274.1430.

#### 3.4.5. Synthesis of 4-(2-Hydroxy-3-((3-methoxyphenyl)amino)propyl)-2-methoxyphenol **3e**

To a suspension of 2-methoxy-4-(oxiran-2-ilmethyl)phenol **2** (0.162 g; 1 equiv) in H_2_O/EtOH 2:1 (2 mL) was added 3-methoxyaniline (0.544 mg; 4.42 mmol), and the resulting mixture was heated at 50 °C for 4 h. Then, water (2 mL) was added, and the resulting mixture was extracted with DCM (2 mL). The organic phase was collected, dried with anhydrous MgSO_4_, and the solvent was evaporated to afford an oil (0.579 g), which was subjected to column chromatography using light petroleum/EtOAc as an eluent of increasing polarity to give compound **3e** as a brown oil (0.098 g; 0.32 mmol; 36%). Rf = 0.35 (ethyl acetate/light petroleum 1:1). ^1^H-NMR δ_H_ (CDCl_3_, 400 MHz): 7.08 (t, J = 8.0 Hz, 1H, Ar-H), 6.86 (d, J = 8 Hz, 1H, Ar-H), 6.73–6.71 (m, 1H, Ar-H), 6.30 (dd, J = 8 Hz, 1.6 Hz, 1H, Ar-H), 6.24 (dd, J = 8 Hz, 2.4 Hz, 1H, Ar-H), 6.20–6.16 (m, 1H, Ar-H), 4.05–3.99 (m, 1H, CH_2_CH(OH)), 3.84 (s, 3H, OCH_3_), 3.76 (s, 3H, OCH_3_), 3.27 (dd, J = 13.2 Hz, 3.6 Hz, 1H, CH_2_NH), 3.05 (dd, J = 12.8 Hz, 7.6 Hz, 1H, CH_2_NH), 2.83–2.70 (m, 2H, CH_2_CH(OH)) ppm. ^13^C-NMR δ_C_ (CDCl_3_, 100.6 MHz): 160.69 (Ar-Cq), 149.42 (Ar-Cq), 146.57 (Ar-Cq), 144.27 (Ar-Cq), 129.99 (Ar-Cq), 129.91 (Ar-CH), 121.85 (Ar-CH), 114.48 (Ar-CH), 111.81 (Ar-CH), 106.35 (Ar-CH), 102.95 (Ar-CH), 102.88 (Ar-CH), 71.07 (CH), 55.76 (OCH_3_), 54.96 (OCH_3_), 49.32 (CH_2_), 41.08 (CH_2_) ppm. HRMS (ESI-TOF): calcd for C_14_H_24_NO_3_ [M^+^ + H]: 304.1543, found 304.1547.

#### 3.4.6. Synthesis of 4-(2-Hydroxy-3-(4-hydroxy-3methoxyphenyl)propyl)amino)benzonitrile **3f**

To a suspension of 2-methoxy-4-(oxiran-2-ilmethyl)phenol **2** (0.162 g; 0.90 mmol; 1 equiv) in H_2_O/EtOH 2:1 (2 mL) was added 4-aminobenzonitrile (0.528 mg; 4.47 mmol), and the resulting mixture was heated at 50 °C for 37 h. Then, water (2 mL) was added, and the resulting mixture was extracted with EtOAc (2 mL), The organic phase was collected, dried with anhydrous MgSO4, and the solvent was evaporated to afford an oil (0.265 g), which was subjected to column chromatography using DCM/MeOH as an eluent of increasing polarity to give compound **3f** as a dark yellow oil (0.025 g; 0.08 mmol; 9%). Rf = 0.45 (MeOH/DCM 5:95). 1H-NMR δ_H_ (CDCl_3_, 400 MHz): 7.39 (d, J = 8.8 Hz, 2H, Ar-H), 6.87 (d, J = 8.4 Hz, 1H, Ar-H), 6.72–6.69 (m, 2H, Ar-H), 6.58–6.50 (m, 2H, Ar-H), 4.07–4.01 (m, 1H, CH_2_CH(OH)), 3.85 (s, 3H, OCH_3_), 3.31 (dd, J = 12.8 Hz, 7.2 Hz, 1H, CH_2_NHPhe), 3.12 (dd, J = 12.8 Hz and 7.4 Hz, 1H, CH_2_NHPhe), 2.82 (dd, J = 13.6 Hz, 5.2 Hz, 1H, CH_2_CH(OH)), 2.73 (dd, J = 13.6 Hz and 8 Hz, 1H, CH_2_CH(OH)) ppm. ^13^C-NMR δ_C_ (CDCl_3_, 100.6 MHz): 151.20 (Ar-Cq), 146.68 (Ar-Cq), 144.53 (Ar-Cq), 136.33 (Ar-Cq), 133.65 (Ar-CH), 128.83 (Ar-Cq), 121.88 (Ar-CH), 121.33 (Ar-CH), 114.62 (Ar-CH), 112.58 (Ar-CH), 111.72 (Ar-CH), 98.91 (Ar-CH), 70.97 (Ar-CH), 55.87 (OCH_3_), 48.10 (CH_2_), 41.20 (CH_2_) ppm.

### 3.5. Cell Culture

Insect cells (*Sf9*, *Spodoptera frugiperda*) cells were maintained as a suspension culture and cultivated in Grace’s medium with 10% FBS and 1% penicillin/streptomycin, at 28 °C with agitation. Cells were used in experiments while in the exponential phase of growth. On the other hand, HaCaT (human keratinocytes) cells were culture in DMEM supplemented with 10% FBS and 1% penicillin/streptomycin at 37 °C in a humidified atmosphere of 5% CO_2_.

### 3.6. Viability Assessment

For the assessment of viability, a resazurin-based method was used. The *Sf9* and HaCaT cells were plated at a density of 3.0 × 10^4^ and 1.5 × 10^4^ cells/well, respectively, incubated for 24 h, and then exposed to the molecules under study (at 100 µg/mL in Grace’s medium) for 24 h. After this period, a commercial solution of resazurin was added (1:10), and the kinetic reaction of fluorescence increase was monitored at 560/590 nm. For HaCaT and the *Sf9* cells, 30 and 60 min of incubation were used, respectively.

### 3.7. LDH Assay

*The Sf9* cells were cultured at the same density described above for the viability assessment. To assess the release of the stable cytosolic enzyme lactate dehydrogenase (LDH) into the media, 24 h after the incubation of cells with the molecules under study (at 100 µg/mL in Grace’s medium), 50 µL of culture media were removed to a 96-well plate. The LDH released was determined using a CytoTox 96^®^ assay kit (Promega; Madison, WI, USA) according to the manufacturer’s instructions. A lysis solution (LS) was used as a positive control to generate a maximum LDH release (45 min). Absorbances were measured at 490 nm in a Multiskan GO plate reader (Thermo Fisher Scientific; Waltham, MA, USA), and results correspond to the fold increase of absorbance in treated vs. untreated cells of four independent experiments performed in duplicate.

### 3.8. Caspase-like Activity

The *Sf9* cells were plated at the same density described for viability studies and exposed to the molecules under study for the designated time. Generally, the same method described before by some of us was used [37]; however, it has been adapted toward insect cells, as previously reported [21,22]. After the incubation period, caspase-3/7 substrate was added to wells, and cells were incubated for 20 min at 22 °C. The luminescent signal was measured in a microplate reader (Cytation™ 3, BioTek, Winooski, VT, USA), and three independent experiments were performed in duplicate. Then, to normalize the results, DNA quantification was performed in a triplicate pool using a Qubit™ 1X dsDNA HS Assay Kit according to a previously described procedure [38] and manufacturer’s instructions.

### 3.9. Statistical Analysis

For biological assays, the Shapiro–Wilks normality test was performed in the data to ensure that it followed a normal distribution. Comparison between the means of controls and each experimental condition was performed using one-way ANOVA. Outliers were identified by the Grubbs’ test. Data were expressed as the mean ± standard deviation (SD) of at least three independent experiments. GraphPad Prism 7.0 software was used, and values were considered statistically significant with a *p* < 0.05.

### 3.10. Molecular Docking and Inverted Virtual Screening Studies

To identify possible molecular targets of the amino alcohols derived from eugenol, an inverted virtual screening protocol was applied. A search on Scopus was performed for papers describing virtual screening (VS) studies involving targets and molecules with insecticidal activity using the keywords: “virtual screening” and “pesticides”. The selection criteria were relevance of the target and year of publication. In the 18 studies found, 23 PDB structures were identified and downloaded, enabling the creation of a structural database of putative insecticide targets. These are listed in Table S3.

The 23 PBD structures were prepared for docking starting using the Autodock Vina plugin for Pymol [39]. Crystallographic waters were removed. Then, the crystallographic ligands were saved in separate files and used as reference for active site coordinates as well as for validation in the re-docking steps. In the absence of ligands, the active site coordinates were based on the most important residues described in the literature. Re-docking was used to evaluate the ability of the docking software to reproduce the geometry and orientation of the crystallographic pose, as well as the quality of the docking protocol, and to optimize the docking protocol.

The docking programs/scoring functions used were GOLD [40] (PLP, ASP, ChemScore, and GoldScore scoring functions), and AutoDock Vina [41]. With each docking program/scoring function, the protocol was optimized for each protein target, to minimize the RMSD in the docking predictions of the reference ligand in re-docking, by comparison with the crystallographic structure of the corresponding complex.

The optimized parameters for each program/scoring function were as follows: Vina-docking box position, docking box dimension, exhaustiveness; GOLD (PLP, ASP, ChemScore, GoldScore)-binding pocket center, docking region radius, search efficiency, number of runs. The final optimized conditions were used for the subsequent stages. Structures for the two eugenol amino alcohol derivatives with the highest insecticide activity were prepared for docking using Datawarrior [42] and OpenBabel [43] and were docked into each structure with the optimized protocol with all the five scoring functions. A ranked list of most likely targets was prepared based on the average scores obtained for each target with the different scoring functions.

### 3.11. Molecular Dynamics Simulations and Free Energy Calculations

Molecular dynamics simulations were performed using the Amber18 software (University of California, San Francisco, USA) for the two compounds identified from the experimental studies to have the highest insecticide activity (compounds **3d** and **3f**), which are bound to the two most promising targets identified from the inverted virtual screening study (odorant binding protein 1–3KIE and acetylcholinesterase-1QON). Since 1QON presented a gap in the structure, a homology model was generated using SWISS-MODEL [44]. A total of 1466 templates were found to match the original sequence, but only the top 50 were used to build the model (Figure S5 in Supplementary Information).

Models for the MD simulations were prepared starting from the pose predicted for these complexes in the docking experiments during the inverted virtual screening protocol with GOLD/PLP and treated with the Leap module of AMBER [45]. The protein targets were described with the ff14SB force field [46], while the eugenol derivatives were parameterized using ANTECHAMBER, with RESP HF/6-31G (d) charges calculated with Gaussian16 [47] and the General Amber Force Field (GAFF) [48]. The overall charge on the system was neutralized through the addition of counter-ions (Na^+^ or Cl^−^), and the systems were placed in TIP3P water boxes with a minimum distance of 12 Å between the protein surface and the side of the box.

In order to remove the clashes, the systems were submitted to four consecutive minimizations stages, which were followed by an equilibration and production. In the first four minimization stages, the procedure was applied to (1) water molecules (2500 steps); (2) hydrogens atoms (2500 steps); (3) side chains of all the amino acid residues (2500 steps); and (4) the full system (10,000 steps). After the complete minimization, the systems were equilibrated by a procedure, which was divided into two stages: in the first stage, NVT ensemble, the systems were gradually heated to 298 K using a Langevin thermostat at constant volume (50 ps); in the second stage, the density of the systems was further equilibrated at 298 K (subsequent 50 ps). Finally, the productions runs were performed during 100 ns. Production was executed with an NPT ensemble at constant temperature (298 K, Langevin thermostat) and pressure (1 bar, Berendsen barostat), with periodic boundary conditions. An integration time of 2.0 fs using the SHAKE algorithm was used to constrain all covalent bonds involving hydrogen atoms. The nonbonded interactions were cut off at 10 Å throughout the entire molecular simulation procedure. The final trajectories were analyzed in terms of RMSD to obtain confirmation that both systems were well equilibrated after the initial 10 ns. The last 90 ns of the simulation were considered for hydrogen bonding analysis, and cluster analysis of the conformations was generated. This overall procedure has been previously used with success in the treatment of several biomolecular systems [49,50,51,52,53,54,55,56,57].

The Molecular Mechanics-Generalized Born Surface Area (MM-GBSA) method [27] was applied to estimate the binding free energies of compounds **3d** and **3f** to the odorant binding protein 1 and to acetylcholinesterase, considering a salt concentration of 0.100 mol.dm^−3^. In addition, the energy decomposition method was employed to estimate the contribution of all the amino acid residues for each of these binding free energies. From each MD trajectory, a total of 1400 conformations taken from the last 70 ns of simulation were considered for the MM-GBSA calculations.

### 3.12. Nanoencapsulation Studies

The most active compound against the *Sf9* cells, compound **3f**, was encapsulated in liposomes composed of the phospholipids 1,2-dimyristoyl-*sn*-glycero-3-phospho- (1′-*rac*-glycerol) (sodium salt) (DMPG) and 1,2-dipalmitoyl-*sn*-glycero-3-phosphocholine (DPPC), which are either composed of DMPG (100%) or DPPC/DMPG (1:1). The liposomes (2 mM total lipid concentration) were prepared by the ethanolic injection method [58] above the transition temperature of each lipid, as previously described [21]. The compound (at an initial concentration of 6.7 × 10^−4^ M) was encapsulated by co-injection with the ethanolic lipid solution. The size, polydispersity, and zeta potential of compound-loaded liposomes were measured in a Litesizer 500 Dynamic Light Scattering apparatus from Anton Paar (Anton Paar GmbH, Graz, Austria).

The encapsulation efficiency (EE%) was determined as previously reported [21] and calculated through the Equation (1):

(1) $$EE\%=\frac{\left(C_{total\;compound}-C_{free\;compound}\right)}{C_{total\;compound}}\times 100$$

using a calibration curve of fluorescence intensity vs. concentration (C), taking advantage of the fluorescence emission of the compound.

The compound release was followed during 24 h at 20 °C and 35 °C, below and above the phase transition temperature of DMPG. The Weibull model (a distribution function) was used to study the transport mechanism involved in the compound release [36], being expressed in terms of the compound fraction accumulated (m) in solution at time t (Equation (2)):

(2) $$m=1-\exp\left[\frac{-{\left(t-T_{i}\right)}^{b}}{a}\right]$$

where a is a scale parameter that defines the timescale of the process, $T_{i}$ represents the latency time of the release process (often being zero), and b is a formal parameter that characterizes the type of curve (*b* = 1 is exponential; *b* > 1 is sigmoid, with ascendant curvature delimited by an inflection point; and *b* < 1 is parabolic, displaying high initial slope and a consistent exponential character).

## 4. Conclusions

A series of β-amino alcohols were prepared by reaction of eugenol epoxide with various aliphatic and aromatic amines. The obtained eugenol derivatives were subjected to biological activity evaluation in the *Sf9* cell line, in comparison with the corresponding precursors, in order to evaluate their application as potential natural based insecticides.

We identified that the three derivatives bearing a terminal benzene ring, either substituted or unsubstituted, were those showing higher potency, in some cases higher than the benchmark used. We further clarified that the molecules were eliciting their effect by triggering organized cell death, and they were selective for insect cells.

Inverted virtual screening studies with five independent methods suggest that these molecules display their insecticide activity most likely by targeting the insect acetylcholinesterase and/or the insect odorant binding proteins. Molecular dynamics simulations and free energy calculations confirm that these two molecules bind strongly to both targets forming very stable complexes with well-defined molecular interactions that are maintained through time.

Nanoencapsulation studies allow obtaining very reasonable encapsulation efficiencies and a controlled release. Liposomes of DMPG provide a temperature-sensitive compound release, which is more effective than the DPPC/DMPG (1:1) formulation.

## Figures and Tables

**Table 1:** Average scores of the amino alcohol derivatives of eugenol obtained for all PDB structures with the five different scoring functions and overall ranking of the most likely protein targets for interaction.

Target	PDB	PLP	ASP	ChemScore	GoldScore	Vina	Overall Ranking
Ecdysone receptor	1R20	68.82	36.26	30.28	57.54	−7.40	5
	1R1K	74.97	35.04	35.86	56.38	−8.60	
Chitinase	3WL1	75.87	50.28	30.745	63.06	−8.05	4
	3WQV	73.47	45.81	31.21	59.7	−8.15	
β-*N*-acetyl-D-hexosaminidase OfHex1	3NSN	78.01	57.04	36.30	70.90	−7.10	3
	3OZP	72.91	51.61	31.11	67.35	−8.05	
*N*-Acetylglucosamine-1-phosphate uridyltransferase (GlmU)	2V0K	65.88	30.02	25.93	55.305	−7.15	13
	2VD4	58.64	28.58	25.97	46.5	−6.20	
Acetylcholinesterase (AChE)	1QON	89.26	58.71	40.87	71.41	−8.90	2
	4EY6	79.52	51.20	39.12	62.63	−8.45	
	1DX4	85.66	51.32	37.48	67.86	−8.70	
Prophenoloxidase (PPO)	3HHS	77.89	40.86	31.48	62.17	−6.85	6
*p*-Hydroxyphenylpyruvate dioxygenase	6ISD	70.33	37.53	31.03	55.00	−7.35	9
Voltage-gated sodium channel	6A95	68.72	31.27	28.95	57.86	−6.75	12
Octopamine receptor	4N7C	57.07	37.56	31.60	61.72	−5.55	11
Sterol carrier protein-2 (HaSCP-2)	4UEI	65.70	37.22	31.64	50.255	−7.40	10
Peptide deformylase	5CY8	76.49	33.49	29.60	65.65	−7.75	7
α-Esterase-7	5TYJ	68.31	39.23	33.26	53.04	−6.95	8
	5TYP	68.54	42.26	32.22	55.565	−6.95	
Odorant Binding Protein	5V13	86.69	52.52	41.24	66.46	−6.90	1
	2GTE	79.14	41.46	38.55	69.11	−7.55	
	3N7H	82.54	44.31	36.01	69.11	−6.90	
	3K1E	91.51	48.43	41.90	74.58	−9.30	

**Table 2:** Average protein and ligand RMSD values (Å), ligand RMSD (Å), average ligand SASA (Å), percentage of potential ligand SASA buried, and average number of ligand–target hydrogen bonds obtained from the MD simulations. ΔG binding energy determined using MM/GBSA and per-residue decomposition, which were calculated for the last 90 ns of the simulation.

		Average RMSD of the Complex (Å)	Average RMSD of the Ligand (Å)	Ligand SASA (Å^2^)	Percentage of Potential Ligand SASA Buried (%)	Average Number H-bonds	ΔG_bind_ (kcal/mol)	Main Contributors
AChE	**3d**	4.6 ± 0.6	1.6 ± 0.4	59.6 ± 16.9	88	0.2 ± 0.1	−18.3 ± 0.1	Trp83 (−2.4 ± 0.8) Tyr370 (−1.3 ± 0.4) His480 (−1.3 ± 0.6)
	**3f**	3.1 ± 0.2	1.4 ± 0.2	36.8 ± 10.2	93	0.5 ± 0.6	−28.2 ± 0.2	Tyr370 (−2.4 ± 0.1) Tyr374 (−2.5 ± 0.8) Trp83 (−1.9 ± 0.4)
OBP	**3d**	2.2 ± 0.3	1.7 ± 0.3	27.2 ± 10.4	95	0.2 ± 0.5	−31.7 ± 0.2	Leu67 (−2.5± 0.5) Trp105 (−2.1± 0.4) Ala79 (−1.7± 0.5)
	**3f**	2.2 ± 0.2	2.1 ± 0.3	33.2 ± 8.5	94	0.9 ± 0.9	−41.6 ± 0.2	Met75 (−2.9 ± 0.4) Trp105 (−2.5 ± 0.4) Phe114 (−1.8 ± 0.8)

**Table 3:** Hydrodynamic diameter (D_h_), polydispersity index (PDI), and zeta (ζ) potential of DMPG (100%) and DMPG:DPPC (1:1) liposomes (SD from three independent measurements).

	D_h_ ± SD (nm)	PDI ± SD	ζ-Potential ± SD (mV)
DMPG (100%)	196 ± 10	0.245 ± 0.007	−43.8 ± 1.8
DMPG:DPPC (1:1)	223 ± 17	0.264 ± 0.014	−55.2 ± 2.4

**Table 4:** Encapsulation efficiency (EE%) of compound **3f** in DMPG (100%) and DMPG/DPPC (50:50) liposomes and concentration of encapsulated compound.

	EE (%)	Encapsulated Concentration (M)
DMPG (100%)	65 ± 7	4.39 × 10^−4^ M
DMPG:DPPC (50:50)	92 ± 1	6.16 × 10^−4^ M

## Data Availability

Not applicable.

## Supplementary Materials

The following are available online, Figure S1: Protein and ligand RMSD (Å) of the AChE and OBP–ligand complexes, Figure S2: Percentage of the potential solvent accessible surface area of the ligands that is buried by the protein targets evaluated, Figure S3: Number of ligand–target hydrogen bonds formed during the simulations for compound **3d** and **3f** when complexed with AchE and OBP, Figure S4: Fitting of the release profiles to the Weibull model, Figure S5: Homology model built for 1QON, Table S1: Docking scores for compounds **3d** and **3f** in complex with human and insect AChE, Table S2: Parameters of the Weibull model for the release of compound **3f** from liposomes and corresponding coefficients of determination (R^2^),Table S3: List of targets selected for the inverted virtual screening study.
