# Supplementary material for: Amino Alcohols from Eugenol as Potential Semisynthetic Insecticides: Chemical, Biological, and Computational Insights
Source: Molecules. 2021 Oct 31;26(21):6616. doi: 10.3390/molecules26216616 (PMC8587747; doi:10.3390/molecules26216616)
Supplement: Supplementary file 1 [file molecules-26-06616-s001.zip › molecules-1405594-supplementary.pdf]

Supplementary Materials

Amino alcohols from eugenol as potential semisynthetic insecticides: chemical, biological and computational insights

Renato B. Pereira [1,†], Nuno F. S. Pinto [2,†], Maria José G. Fernandes [2], Tatiana F. Vieira [3,4], Ana Rita O. Rodrigues [5], David M. Pereira [1], Sérgio F. Sousa [3,4], Elisabete M. S. Castanheira [5], A. Gil Fortes [2] and M. Sameiro T. Gonçalves [2,*]

[1] REQUIMTE/LAQV, Laboratory of Pharmacognosy, Department of Chemistry, Faculty of Pharmacy, University of Porto, R. Jorge Viterbo Ferreira, 228, 4050-313 Porto, Portugal; rjpereira@ff.up.pt (R.B.P.); dpereira@ff.up.pt (D.M.P.)

[2] Centre of Chemistry, Department of Chemistry, University of Minho, Campus of Gualtar, 4710-057 Braga, Portugal; nuno_pinto1993@hotmail.com (N.F.S.P.); mjfernandes@quimica.uminho.pt (M.J.G.F.); gilf@quimica.uminho.pt (A.G.F.)

[3] Associate Laboratory i4HB—Institute for Health and Bioeconomy, Faculty of Medicine, University of Porto, 4200-319 Porto, Portugal; segiofsousa@med.up.pt (S.F.S.); tatianafvieira@gmail.com (T.F.V.)

[4] UCIBIO—Applied Molecular Biosciences Unit, BioSIM—Department of Biomedicine, Faculty of Medicine, University of Porto, 4200-319 Porto, Portugal

[5] Centre of Physics of Minho and Porto Universities (CF-UM-UP), University of Minho, Campus of Gualtar, 4710-057 Braga, Portugal; ritarodrigues@fisica.uminho.pt (A.R.O.R.); ecoutinho@fisica.uminho.pt (E.M.S.C.)

* Corresponding author: msameiro@quimica.uminho.pt; Tel.: + 351-253-604-372

† These authors contributed equally to this work.

1. Creation of a Homology Model

The model generated by SWISS-MODEL for 1QON was used in the MD simulations since the gap that was missing from the original structure was distant from the active site.

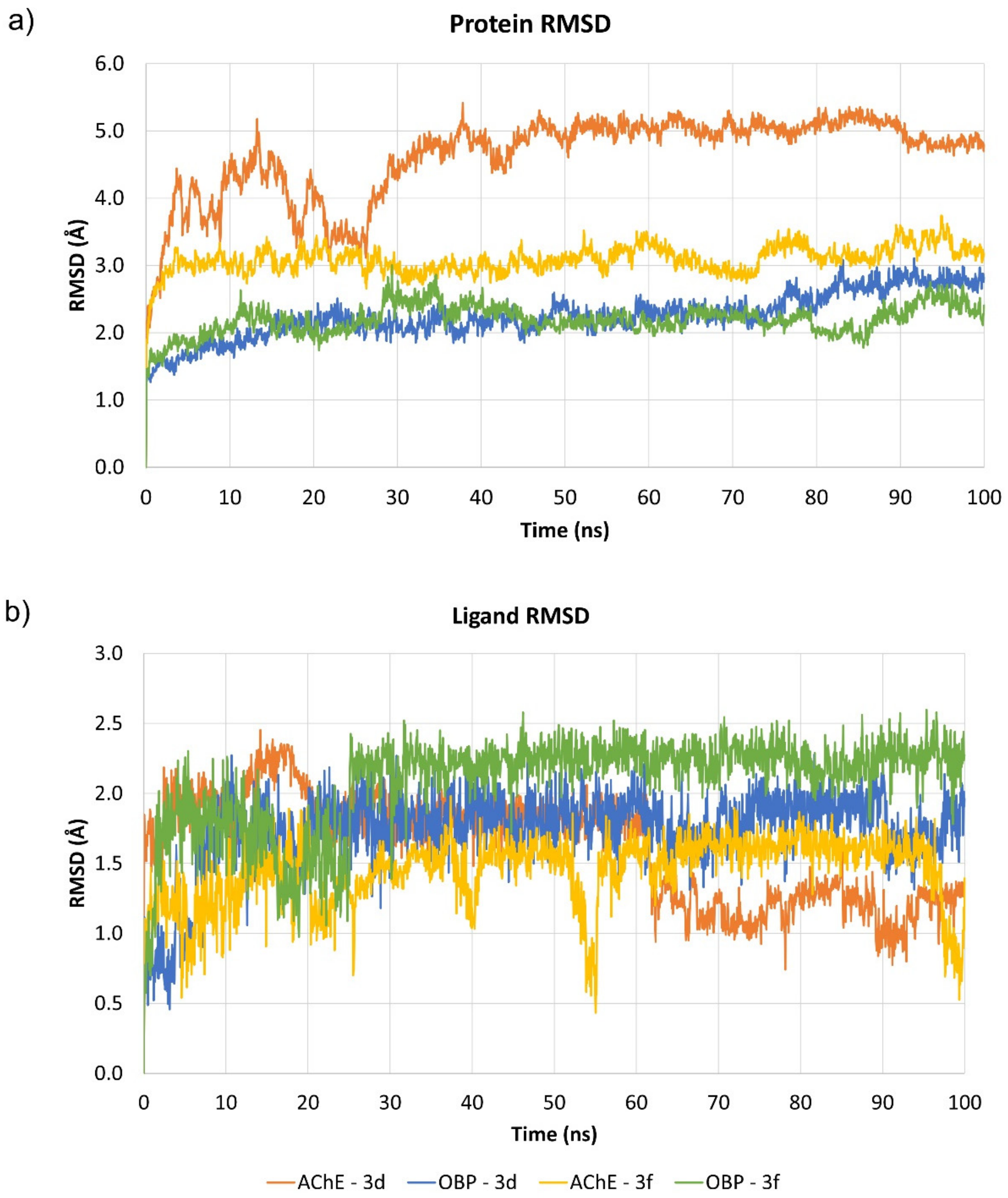

Figure S1. Protein and ligand RMSD (Å) of the AChE and OBP – ligand complexes.

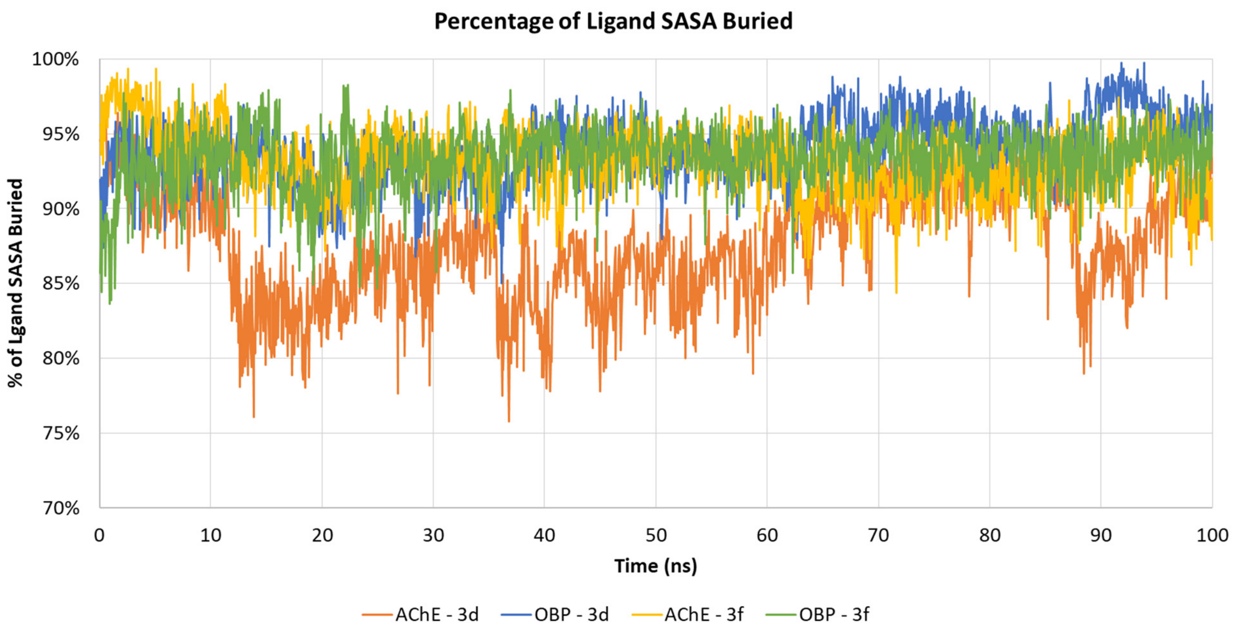

Figure S2. Percentage of the potential solvent accessible surface area of the ligands that is buried by the protein targets evaluated.

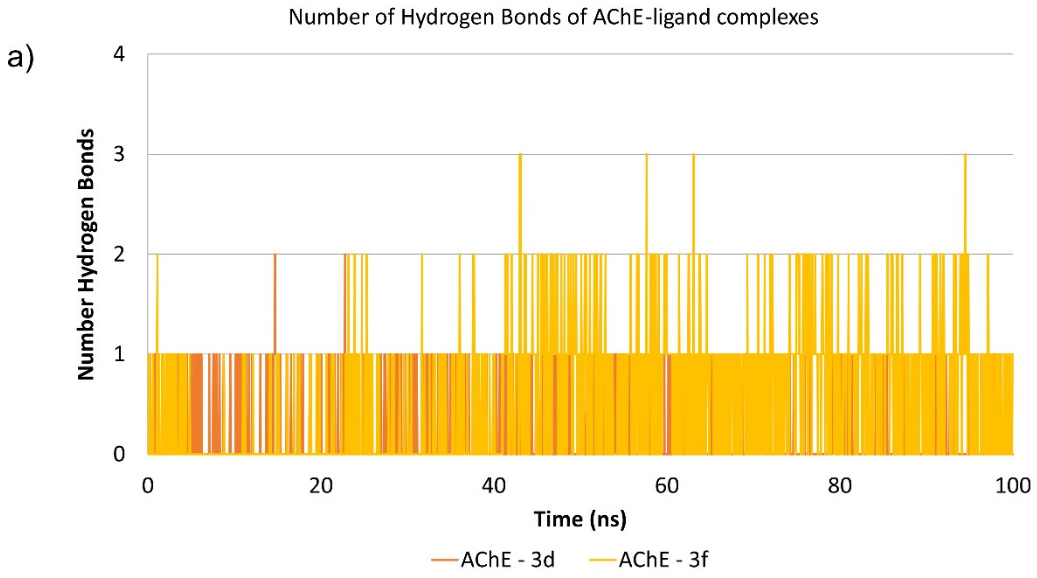

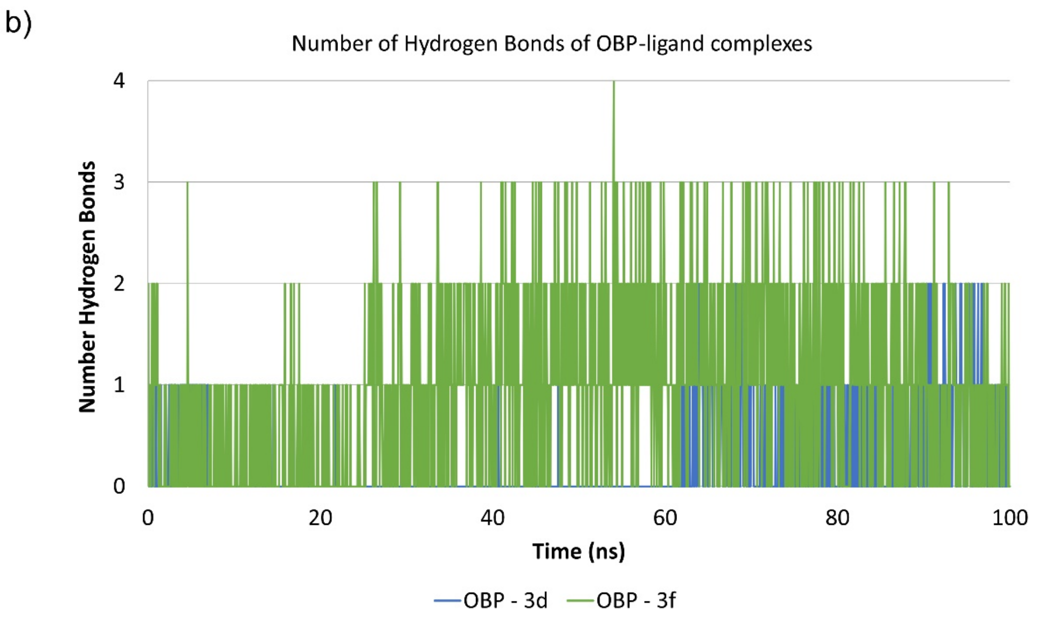

Figure S3. Number of ligand-target hydrogen bonds formed during the simulations for compound **3d** and **3f** when complexed with AchE and OBP.

Table S1. Docking scores for compound **3d** and **3f** in complex with Human and insect AChE.

		PLP	ASP	ChemScore	GoldScore	Vina
Human AChE	**3d**	78.04	50.27	39.38	62.94	-8.2
Insect AChE	**3d**	86.78	57.17	39.08	69.49	-8.9
Human AChE	**3f**	76.74	53.87	32.95	64.08	-8.3
Insect AChE	**3f**	91.73	60.25	24.66	73.33	-8.9

2. Compound release kinetics

Table S2. Parameters of the Weibull model for the release of compound **3f** from liposomes and corresponding coefficients of determination (R^2).

	**T (ºC)**	Y_{max}	b	a	R^2
**DMPG (100%)**	20	39.28	0.67	0.26	0.99
	35	58.56	1.35	0.14	0.99
**DPPC:DMPG (50:50)**	20	14.30	1.16	0.17	0.99
	35	15.45	1.26	0.12	0.98

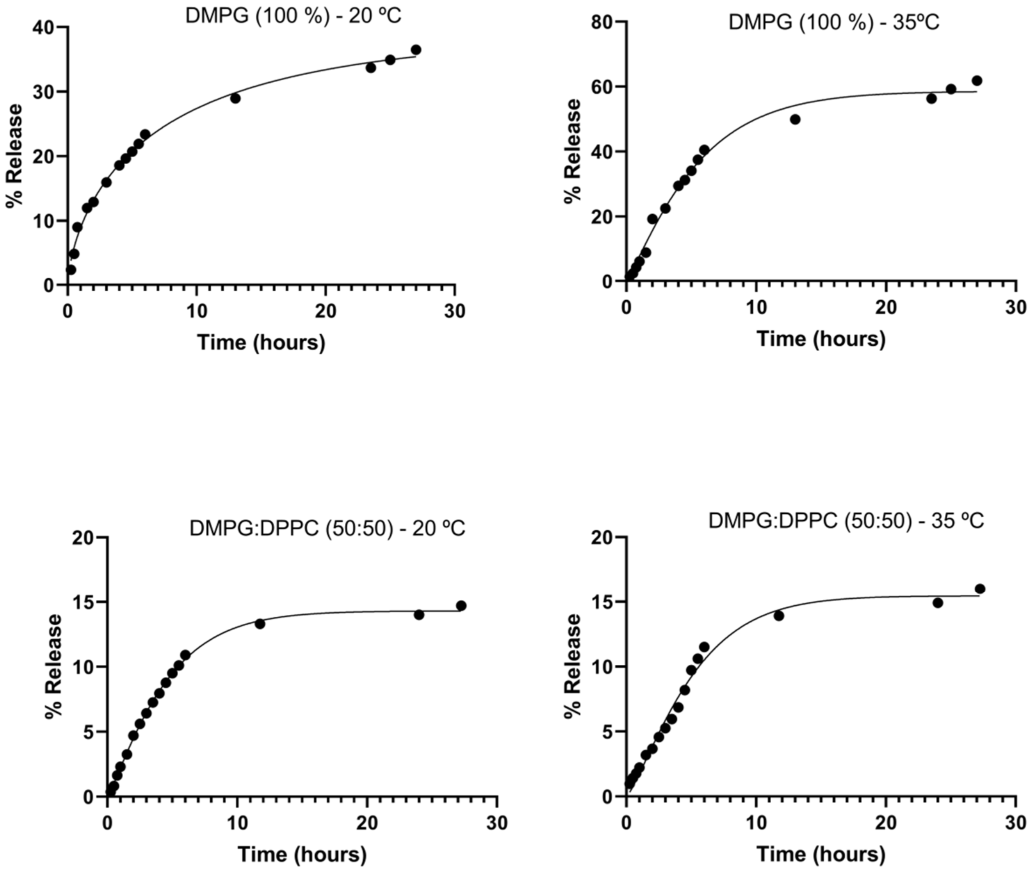

Figure S4. Fitting of the release profiles to the Weibull model.

3. Molecular docking and inverted virtual screening studies

Table S3. List of targets selected for the inverted virtual screening study.

Target	Organism	PDB target	Resolu-tion (Å)	Description	Ref.
**Ecdysone receptor**	*Heliothis virescens*	1R20	3.00	VS based on 1R20 bound to an agonist as a model for the development of a receptor-based pharmacophore model.	1
		1R1K	2.90	VS of 2 million compounds against 1R1K, an ecdysone receptor structure bound to its known ligand ponasterone A.	2
**Chitinase**	*Ostrinia furnacalis*	3WL1	1.77	Pharmacophore-based screening using two crystal structures of chitinases: 3WL1 bound to its reaction product and 3WQV bound to an inhibitor.	3
		3WQV	2.04		
**beta-*N*-acetyl-D-hexosaminidase OfHex1**		3NSN	2.10	VS of the ZINC database to identify OfHex1 inhibitors using 3NSN crystal structure bound to a known inhibitor.	4
		3OZP	2.00	VS of the ZINC data-base targeting 3OZP, a crystal structure of OfHex1 bound to an inhibitor.	5
***N*-Acetyl-glucosamine-1-phosphate uridyl-transferase (GlmU)**	*Xanthomonas oryzae*	2V0K	2.30	Homology model built for docking using 2V0K and 2VD4 as templates. 2V0K crystal structure is bound to its known ligand and 2VD4 is bound to a possible inhibitor.	6
		2VD4	1.90		
**Acetylcholines-terase**	*Aedes aegypti*	1QON	2.72	Search for new molecules with insecticidal activity against *Ae. Aegypti* using acetylcholinesterase crystal structures 1QON and 4EY6 as targets, both bound to possible inhibitors.	7
		4EY6	2.40		
	Drosophila melanogaster	1DX4	2.70	Homology 3D model built for VS using 1DX4 as template. 1DX4 crystal structure is bound to a potent inhibitor.	8
**Prophenol-oxidase (PPO)**	*Manduca sexta*	3HSS	1.97	Crystal structure of a prophenoloxidase from *Manduca sexta.*	9
***p*-Hydroxyphenyl-pyruvate dioxygenase**	*Arabidopsis thaliana*	6ISD	2.40	Development of a receptor-ligand pharmacophore model based on the crystal structure 6ISD bound to a commonly used pesticide. The best model created was then used for VS studies.	10
**Voltage-gated sodium channel**	*Periplaneta americana*	6A95	2.60	Crystallographic structure of a Voltage-gated sodium channel NavPaS bound to a pore blocker, tetrodotoxin (TTX)	11
**Octopamine receptor**	*Blattella germanica*	4N7C	1.75	Crystal structure of Bla g 4, an octopamine receptor, bound to tyramine.	12
**Sterol carrier protein-2 (HaSCP-2)**	*Helicoverpa armigera*	4UEI	Solution NMR	Structure-based VS of a database of commercially available compounds to find potential inhibitors of HaSCP-2. The residues Phe53, Thr128, and Gln131 were selected for the binding cavity.	13

**Peptide deformylase**	*Xanthomonas oryzae*	5CY8	2.38	Docking and VS of a library of 318 phytochemicals. 5CY8 crystal structure is bound to a possible inhibitor.	14
**Alpha-esterase-7 (αE7)**	*Lucilia cuprina*	5TYJ	1.75	Computational design of potent and selective covalent inhibitors of αE7. 5TYJ and 5TYP crystal structures are bound to inhibitors: (3-bromo-5-phenoxylphenyl)boronic acid and (3-bromo-4-methylphenyl)boronic acid respectively.	15
		5TYP	1.88		
**Odorant Binding Protein**	*Aedes aegypti*	5V13	1.84	Search for new molecules with insecticidal activity against *Ae. Aegypti* using a crystal structure of a mosquito juvenile hormone-binding protein, 5V13 bound to its natural hormone.	7
	Drosophila melanogaster	2GTE	1.40	2GTE crystal structure is bound to its natural ligand	16
	Anopheles gambiae	3N7H	1.60	QSAR and docking studies for the rational design of mosquito repellents using the crystal structure 3K1E bound to a polyethylene glycol molecule. 3N7H crystal structure is bound to a commonly used repellent.	17
	Aedes aegypti	3K1E	1.85		

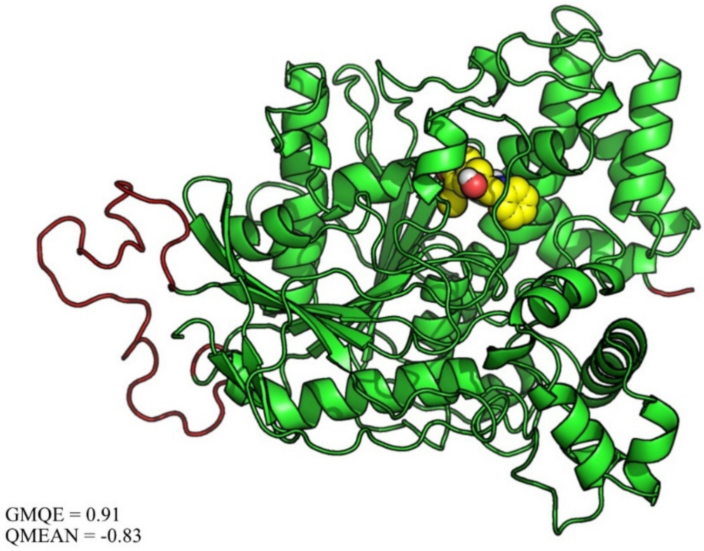

Figure S5. Homology model built for 1QON. Green is the original structure and red represents the loop that was generated by SWISS-MODEL. In yellow is the ligand molecule (**3d**). GMQE - Global Model Quality Estimation, is expressed between 0 and 1 with a higher number meaning higher reliability. QMEAN - provides an estimate of the "degree of nativeness" of the structural features observed in the model. A value of QMEAN around zero indicate a good agreement between the model and experimental structure.

References

1. Hu, X.; Yin, B.; Cappelle, K.; Swevers, L.; Smagghe, G.; Yang, X.; Zhang, L. Identification of novel agonists and antagonists of the ecdysone receptor by virtual screening. *J Mol Graph Model* **2018**, *81*, 77–85.
2. Harada, T.; Nakagawa, Y.; Ogura, T.; Yamada, Y.; Ohe, T.; Miyagawa, H. Virtual screening for ligands of the insect molting hormone receptor. *J Chem Inf Model* **2011**, *51*, 296–305.
3. Dong, Y.; Jiang, X.; Liu, T.; Ling, Y.; Yang, Q.; Zhang, L.; He, X. Structure-based virtual screening, compound synthesis, and bioassay for the design of chitinase inhibitors. *J Agric Food Chem* **2018**, *66*(13), 3351-3357.
4. Liu, J.; Liu, M.; Yao, Y.; Wang, J.; Li, Y.; Li, G.; Wang, Y. Identification of novel potential β-N-acetyl-D-hexosaminidase inhibitors by virtual screening, molecular dynamics simulation and MM-PBSA calculations. *Int J Mol Sci* **2012**, *13*, 4545–4563.
5. Dong, L.; Shen, S.; Xu, Y.; Wang, L.; Yang, Q.; Zhang, J.; Lu, H. Identification of novel insect β-N-acetylhexosaminidase OfHex1 inhibitors based on virtual screening, biological evaluation, and molecular dynamics simulation. *J Biomol Struct Dyn* **2021**, *39*, 1735–1743.
6. Min, J.; Lin, D.; Zhang, Q.; Zhang, J.; Yu, Z. Structure-based virtual screening of novel inhibitors of the uridyltransferase activity of Xanthomonas oryzae pv. oryzae GlmU. *Eur J Med Chem* **2012**, *53*, 150–158.
7. Ramos, R.S.; Costa, J.S.; Silva, R.C.; Costa, G.V.; Rodrigues, A.B.L.; Rabelo, E.M.; Souto, R.N.P.; Taft, C.A.; Silva, C.H.T.P.; Rosa, J.M.C.; Santos, C.B.R.; Macêdo, W.J.C. Identification of potential inhibitors from pyriproxyfen with insecticidal activity by virtual screening. *Pharmaceuticals* **2019**, *12*, 20.
8. Riva, C.; Suzanne, P.; Charpentier, G.; Dulin, F.; Halm-Lemeille, M.P.; Santos, J.S.O. In silico chemical library screening and experimental validation of novel compounds with potential varroacide activities. *Pestic Biochem Physiol* **2019**, 160, 11–19.
9. Fattouch, S.; Raboudi-Fattouch, F.; Ponce, J.V.; Forment, J.V.; Lukovic, D.; Marzouki, N.; Vidal, D.R. Concentration dependent effects of commonly used pesticides on activation versus inhibition of the quince (Cydonia Oblonga) polyphenol oxidase. *Food Chem Toxicol* **2010**, *48*, 957–963.
10. Fu, Y.; Liu, Y.-X.; Kang, T.; Sun, Y.-N.; Li, J.-Z.; Ye, F. Identification of novel inhibitors of p-hydroxyphenylpyruvate dioxygenase using receptor-based virtual screening. *J Taiwan Inst Chem Eng* **2019**, *103*, 33–43.
11. Shen, H.; Li, Z.; Jiang, Y.; Pan, X.; Wu, J.; Cristofori-Armstrong, B.; Smith, J.J.; Chin, Y.K.Y.; Lei. J.; Zhou, Q.; King, G.F.; Yan, N. Structural basis for the modulation of voltage-gated sodium channels by animal toxins. *Science* **2018**, *362*, 1–8.
12. Offermann, L.R.; Chan, S.L.; Osinski, T.; Tan, Y.W.; Chew, F.T.; Sivaraman, J.; Mok, Y.K.; Minor, W.; Chruszcz, M. The major cockroach allergen Bla g 4 binds tyramine and octopamine. *Mol Immunol* **2014**, *60*, 86–94.
13. Cai, J.; Du, X.; Wang, C.; Lin. J.; Du, X. Identification of potential helicoverpa armigera (Lepidoptera: Noctuidae) sterol carrier protein-2 inhibitors through high-throughput virtual screening. *J Econ Entomol* **2017**, 110, 1779–1784.
14. Joshi, T.; Joshi, T.; Sharma, P.; Chandra, S.; Pande. V. Molecular docking and molecular dynamics simulation approach to screen natural compounds for inhibition of Xanthomonas oryzae pv. Oryzae by targeting peptide deformylase. *J Biomol Struct Dyn* **2021**, *39*, 823–840.
15. Correya, G.J.; Zaidman, D.; Harmelin, A.; Carvalho, S., Mabbitta, P.D.; Calaora, V.; James, P.J.; Kotzeg, A.C.; Jackson, C.J.; London, N. Overcoming insecticide resistance through computational inhibitor design. *Proc Natl Acad Sci* **2019**, 116, 21012–21021.
16. Laughlin, J.D.; Ha, T.S.; Jones, D.N.M.; Smith, D.P. Activation of pheromone-sensitive neurons is mediated by conformational activation of pheromone-binding protein. *Cell* **2008**, *133*, 1255–1265.
17. Oliferenko, P.V.; Oliferenko, A.A.; Poda, G.I.; Osolodkin, D.I.; Pillai, G.G.; Bernier, U.R.; Tsikolia, M.; Agramonte, N.M.; Clark, G.G.; Linthicum, K.J.; Katritzky, A.R. Promising aedes aegypti repellent chemotypes identified through integrated QSAR, virtual screening, synthesis, and sioassay. *PLoS One* **2013**, *8*, 2 e64547.
